# Adoption and Attitudes of eHealth Among People Living With HIV and Their Physicians: Online Multicenter Questionnaire Study

**DOI:** 10.2196/16140

**Published:** 2020-04-15

**Authors:** Christine Jacomet, Roxana Ologeanu-Taddei, Justine Prouteau, Céline Lambert, Françoise Linard, Pascale Bastiani, Pierre Dellamonica

**Affiliations:** 1 Infectious Diseases Department Clermont-Ferrand University Hospital Clermont-Ferrand France; 2 Systèmes d'Information – Recherche en Management & Polytech Montpellier University Montpellier France; 3 Delegation Recherche Clinique et Innovation Clermont-Ferrand University Hospital Clermont-Ferrand France; 4 Infectious Disease Department Assistance Publique Hôpitaux de Paris – Sorbonne Université Paris France; 5 Agissons Contre le Sida Nice France; 6 Cote d'Azur University Nice France

**Keywords:** survey, HIV, eHealth, internet for information retrieval, health applications, connected objects, telemedicine, collection of digitized personal information

## Abstract

**Background:**

The development of electronic health (eHealth) has offered the opportunity for remote care provision. eHealth addresses issues for patients and professionals favoring autonomy and compliance, respectively, while fostering closer links both between patients and health care professionals and among health care professionals themselves.

**Objective:**

The aim of this study was to analyze the patterns of use, benefits, and perceived obstacles in eHealth among people living with HIV (PLHIV) and their caring physicians at hospitals.

**Methods:**

An online multicenter observational survey was conducted October 15-19, 2018 in 51 medical units across France by means of self-administered questionnaires to collect sociodemographic and medical data, and perceptions of eHealth. Multiple correspondence analysis followed by mixed unsupervised classification were performed to analyze data of the respondents.

**Results:**

A total of 279 PLHIV and 219 physicians responded to all parts of the questionnaire. Three groups of PLHIV were identified based on multivariate analysis. Group 1 comprised “eHealth believers” (121/279, 43.4%), who were more frequently above 60 years old and more likely to be receiving treatments other than antiretrovirals. Group 2, the “technology skeptics” (86/279, 30.8%), comprised more women with at least one child. Group 3, the “internet adopters” (72/279, 25.8%), were more frequently under 49 years of age, men who have sex with men, and more likely to use mobile apps for obtaining wellness/health information and related subjects. Three groups of physicians also emerged. Group 1 comprised those “strongly confident in eHealth” (95/219, 43.4%), who more frequently used mobile apps for wellness/health information and were more likely to accept prescription assistance software. Group 2 comprised physicians “strongly opposed to eHealth” (80/219, 36.5%), frequently asserting that eHealth challenges confidentiality. Group 3 were “open to eHealth” (44/219, 20.1%), comprising a higher proportion of infectious disease specialists, and were more likely to believe that medical apps are useful for patient education and information. No link was found between the groups of PLHIV and physicians.

**Conclusions:**

The literature on eHealth mainly classifies people as enthusiasts and skeptics; however, we identified a third profile among both PLHIV and physicians, albeit without a direct link between them. For PLHIV, this third group is attentive to eHealth for improving their health condition, and for physicians, this group considers eHealth to offer benefits to patients and their own practice.

## Introduction

The World Health Organization defines eHealth (electronic health) as the application of information and communication technologies to all activities connected to health [1]. As eHealth offers remote care provision, such technologies can favor patient autonomy and compliance, while fostering closer links between patients and health care professionals, as well as among health care professionals themselves [2]. Data processing via computers, connected devices (eg, mobile phones, smartphones, tablets) [3-6], and connected objects has witnessed exponential development in recent years involving a broad diversity of actors [7].

The emergence and spread of HIV infection paralleled the development of eHealth within a short time period. In the last three decades, there has been a rise in freely accessible online journals, eLearning, and massive open online courses, which now supersede the printed page as the primary source of scientific knowledge for health care professionals [8]. Moreover, the development of new channels of communication between patients and their health care professionals poses a threat to medical confidentiality [9]. Improvements in the traceability and storage of medical data are raising ethical questions [10], while the economic value of such information has increased. In response, the General Data Protection Regulation was approved on April 14, 2016 by the European Parliament, and came into effect in France on May 25, 2016, which restricts the use of such data [11].

At the same time, France devoted 11% of its gross domestic product (GDP; 198.5 billion Euros [214 billion USD]) to health [12]. Health costs are increasing faster than the GDP (+1.6%), calling for an economic and ethical review of public health spending. The French health care system must meet many challenges, including the increased prevalence in chronic illness (estimated to affect 37% of the French population in 2014 [13]), an aging society, medically deprived areas, shortage of caregivers, and increased costs of care. One approach to maintaining quality health care is through reorganization. The use of eHealth tools to follow patients remotely and coordinate their care teams should be a step in this direction.

Analysis of the literature in the field of eHealth, and in particular of connected objects [14], considering current trends, utility, and limits shows that eHealth technologies are perceived in two distinct, but specific, ways. The first is clear enthusiasm and especially high hopes for new health technologies, and the other is criticism of the development of these technologies, in some cases questioning their usefulness and highlighting the potential danger to confidentiality, while destroying the physician-patient relationship through the dehumanization of care.

In the present study, we analyzed the behaviors, benefits, and barriers perceived by people living with HIV (PLHIV) and their physicians via online self-administered questionnaires (for details on questionnaire development, see personal communication with the first author CJ) to determine whether any additional profiles of eHealth perception exist besides these two sharply opposing viewpoints, and whether there is a link between the perception profiles of PLHIV and physicians.

## Methods

### Recruitment and Questionnaire Design

We conducted a multicenter observational single-week survey involving all of the patients infected with HIV who were consulted during the week of October 15-19, 2018 in French hospitals via regional coordination structures. The patients were involved in the design, implementation, reporting, and dissemination of this survey.

The inclusion criterion was aged over 18 years. Exclusion criteria were inability to reply to the questionnaire, inability to speak French, and refusal to participate. This observational survey was compliant with the MR003 reference specification [15]. The protocol was filed with the French data protection agency (CNIL, No. M18009).

### Data Collection

Study data were collected and managed using Research Electronic Data Capture (REDCap) electronic data capture tools hosted at the teaching hospital Centre Hospitalier Universitaire (CHU) de Clermont-Ferrand (Clermont-Ferrand, France) [16,17]. REDCap is a secure, web-based software platform designed to support data capture for research studies, providing an intuitive interface for validated data capture, audit trails for tracking data manipulation and export procedures, automated export procedures for seamless data downloads to common statistical packages, and procedures for data integration and interoperability with external sources. Both patients and health care professionals generated their own anonymous data using the REDCap app created for this survey. Access to the survey was provided through a QR code or internet link, followed by entry of an access code. The data were accessible only to the staff of the Clinical Research Department of CHU Clermont-Ferrand, which takes responsibility for their security and confidentiality.

### Data Analysis

All statistical analyses were performed using Stata software (version 13, StataCorp, College Station, TX, USA) and R 3.3.3 software (R Foundation for Statistical Computing, Vienna, Austria). All tests were two-sided with a type I error set at .05. Baseline characteristics (physicians and patients) are presented as frequencies and associated percentages for categorical parameters, and as mean (SD) or median (interquartile range) for continuous variables, according to the data distribution.

Multiple correspondence analysis followed by mixed unsupervised classification (k-means clustering applied to the partition obtained from an ascending hierarchical classification using Ward distance) were implemented to (i) examine the relations between the modalities of the variables and (ii) determine PLHIV profiles (clusters of individuals sharing closely similar characteristics). For these analyses, the variables were chosen according to univariate results, clinical relevance, and the data distribution (parameters always present or always absent were not considered), which included sociodemographic and medical characteristics of PLHIV, patterns of behavior and opinions regarding information retrieval via the internet and social media, representations of using apps and connected objects, and opinions on distance consultation and on collection of personal health data. The association between the groups obtained and their characteristics were compared by the Chi-squared test.

The same analyses were performed for the physicians with the same variables. The questionnaire for physicians also addressed perceptions of their patients’ use of eHealth provisions.

Finally, the clinically identified groups of PLHIV were compared with those of physicians by a Chi-squared test to examine similarities between profiles.

## Results

### Overall Response

The survey was conducted in 51 medical units throughout France after consent was obtained from 255 physicians who consulted with 1377 patients during the study period. Among these patients, 144 were not eligible, 395 refused to take part in the study, and 838 agreed to participate and were given access to the online survey. Overall, 325/838 (38.8%) patients followed in 46 centers, including 191 (58.8%) at teaching hospitals, filled out the online questionnaire; 117 (36.0%) of the patients were residents of the Ile-de-France region. Of the patients, 279 answered all of the questions of interest, and their questionnaires were ultimately included in the analysis. These patients were comparable to the 46 excluded patients with regard to the variables used for analyzing patterns of use and behavior for information retrieval via the internet and social media, use of apps and connected objects, distance consultations, and collection of personal health data.

Nearly all the physicians who replied to the questionnaire (219/227, 96.5%) were included in the factorial analysis.

**Figure 1:**
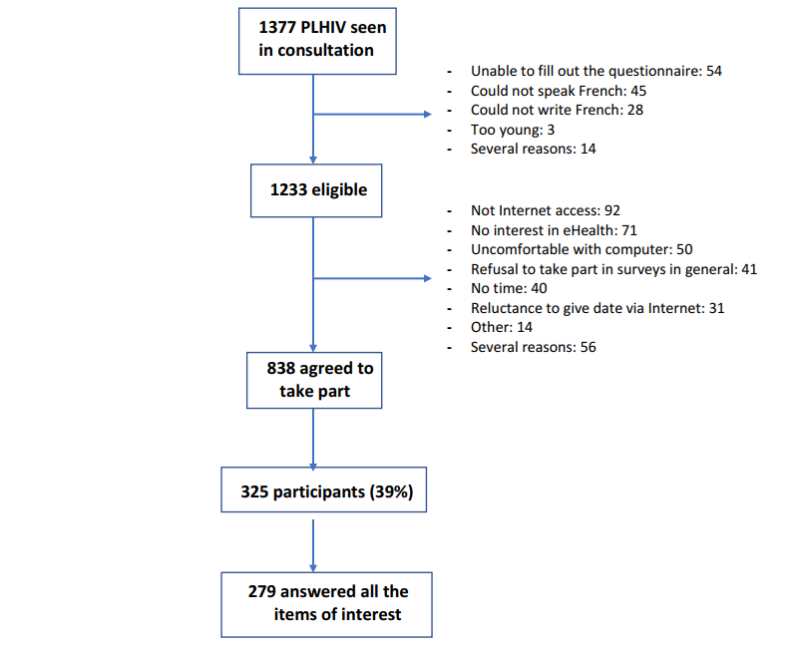
Flowchart of the 279 people living with HIV (PLHIV) who answered all items of interest.

The reasons for noninclusion and refusal are given in Figure 1.

### Sociodemographic and Clinical Characteristics of Patients and Physicians

Table 1 provides a summary of the sociodemographic characteristics, and Table 2 summarizes the key clinical and care path characteristics of the patients included in the analysis.

The 279/325 (85.8%) PLHIV who responded to all of the items included in the analysis (no missing data) were mostly middle-aged males born in France. Half of them lived with a partner. The majority had high school or higher education, and were approximately evenly split between those with a stable job and those in a precarious employment situation. They had been living with HIV for a mean of 17 years and had been receiving antiretroviral treatment for a mean of 14 years with an undetectable viral charge (<50 copies/ml) in most cases and an average CD4 count of 620/mm^3^; less than half of the patients were also receiving associated treatments. Most of the patients consulted their general practitioner 1-3 times per year and their HIV specialist twice a year, and less than a third did not consult any other specialist physicians. At the time the questionnaire was filled out, the average fitness of the patients was self-evaluated to be relatively high on a visual analog scale.

Of the 255 physicians who agreed to take part in the survey, 227 (89.0%) answered the medical questionnaire, and 219 (85.9%) answered all of the questions of interest for the analysis. The majority worked full-time at the hospital, mainly in an infectious disease department; the other physician characteristics are summarized in Table 3.

**Table 1:** Sociodemographic characteristics of people living with HIV (N=279).

Characteristic		Value
Age (years), mean (SD)		53 (12)
**Gender, n (%)**		
	Male	199 (71.3)
	Female	80 (28.7)
Live with a partner, n (%)		142 (50.9)
**Sexual orientation, n (%)**		
	Heterosexual	127 (45.5)
	Homosexual	120 (43.0)
	Other	11 (3.9)
	Decline to reply	21 (7.5)
At least one child, n (%)		119 (42.7)
Country of birth=France, n (%)		218 (78.1)
Region of birth=Paris/Ile-de-France, n (%)		55 (19.7)
Region of residence=Paris/Ile-de-France, n (%)		89 (31.9)
High school or above education level, n (%)		184 (65.9)
**Occupation, n (%)**		
	Stable job	130 (46.6)
	Retired	61 (21.9)
	Invalid	35 (12.5)
	Job-seeker	29 (10.4)
	Other	24 (8.6)
**Precarity**		
	EPICES^a^ precarity score, median (IQR)	25 (15-46)
	Precarious, n (%)	127 (45.5)
**Meeting places, n (%)**		
	Bars, clubs without sex	81 (29.0)
	Sex clubs	40 (14.3)
	Through geolocating dating sites	57 (20.4)

^a^EPICES: Evaluation of Precarity and Inequalities in Health Examination Centers.

**Table 2:** Clinical and care path characteristics of people living with HIV (N=279).

Characteristic		Value
**Medical characteristics**		
	Last viral load undetectable, n (%)	255 (91.4)
	Last CD4 count (mm^3^), median (IQR)	600 (400-842)
	Years since HIV infection detected, mean (SD)	17 (10)
	Years of antiviral treatment, mean (SD)	14 (8)
**Tobacco use, n (%)**		
	Active	76 (27.2)
	Nonsmoker or exsmoker	203 (72.8)
Alcohol consumption more than once a week, n (%)		136 (48.7)
**Consumption of recreational drugs, n (%)**		
	Active or former user	55 (19.7)
	Non-user	224 (80.3)
Lipodystrophy, n (%)		56 (20.1)
**Associated treatments, n (%)**		
	Presence	123 (44.1)
	Anti-HBP^a^	57 (20.4)
	Psychiatric	43 (15.4)
	Cardiovascular	27 (9.7)
	Anti-diabetes	24 (8.6)
	Hyperlipidemia	15 (5.4)
	Bone and joints	15 (5.4)
	Neurological	13 (4.7)
	Hepatitis B or C	9 (3.2)
	Renal	8 (2.9)
	Cancer	5 (1.8)
**Care path, n (%)**		
	Follow up in teaching hospital	239 (85.7)
	No consultation in general medical practice	39 (14.0)
	One to three consultations in general medical practice	163 (58.4)
	Four or more consultations in general medical practice	77 (27.6)
	One or more HIV-specific consultations per year	159 (57.0)
	Three or more HIV-specific consultations per year	120 (43.0)
	No other specialized consultations	81 (29.0)
	One to three other specialized consultations	150 (53.8)
	Four or more other specialized consultations	48 (17.2)
Fitness VAS^b^, mean (SD)		78 (19)

^a^HBP: high blood pressure.

^b^VAS: visual analog scale (0-100).

**Table 3:** Sociodemographic characteristics of physicians (N=219).

Sociodemographic characteristic		Value
Age (years), mean (SD)		48 (10)
**Gender, n (%)**		
	Male	94 (42.9)
	Female	125 (57.1)
**Medical specialty, n (%)**		
	Infectious diseases	158 (72.1)
	General practitioner	37 (16.9)
	Internal medicine	13 (5.9)
	Dermatology	4 (1.8)
	Hematology	2 (0.9)
	Gastroenterology	1 (0.5)
	Geriatrics	1 (0.5)
	Immunology	1 (0.5)
	Psychiatry	1 (0.5)
	Public health	1 (0.5)
**Hospital practice, n (%)**		
	Full time	159 (72.6)
	Part time	60 (27.4)
Department of practice in Paris/Ile-de-France, n (%)		80 (36.5)

### Patient Profiles on eHealth Perceptions

#### Overall Response

**Figure 2:**
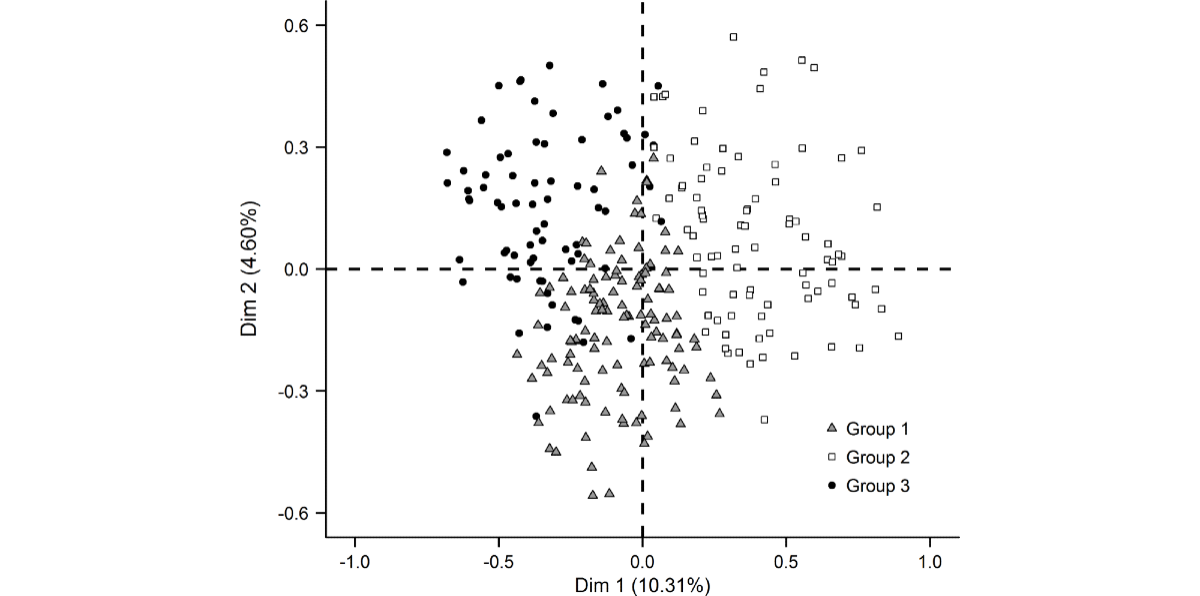
Factorial analysis of people living with HIV by sociodemographic and medical characteristics, and their answers to 113 questions concerning eHealth: searching for information on the internet and social media, collection of digitized personal information, and mHealth apps and connected objects for health/wellness.

The analysis was focused on the 279/325 PLHIV for whom there were no missing data among the variables selected for the analysis. Multiple correspondence analysis identified 3 groups (G) of patients: G1 (121/279, 43.4%), termed “eHealth believers;” G2 (86/279, 30.8%), termed “technology skeptics;” and G3 (72/279, 25.8%), termed “internet adopters” (Figure 2).

Their main epidemiological, medical, and eHealth perception characteristics are summarized by group in Table 4 and Table 5 (see Multimedia Appendix 1 for detailed results). Only statistically different variables are presented.

**Table 4:** Sociodemographic and medical characteristics, internet and social media, app use, and connected objects for three groups of people living with HIV obtained by mixed unsupervised classification.

Characteristic		Total (N=279)	Group 1 (n=121)	Group 2 (n=86)	Group 3 (n=72)
**Sociodemographic characteristics, n (%)**					
	Aged <49 years	106 (38.0)	33 (27.3)	26 (30.2)	47 (65.3)
	Aged >60 years	79 (28.3)	48 (39.7)	25 (29.1)	6 (8.3)
	Male	199 (71.3)	90 (74.4)	51 (59.3)	58 (80.6)
	MSM^a^	120 (43.0)	53 (43.8)	21 (24.4)	46 (63.9)
	At least one child	119 (42.7)	50 (41.3)	48 (55.8)	21 (29.2)
	Higher education	184 (65.9)	73 (60.3)	51 (59.3)	60 (83.3)
	Frequent use of geolocating dating sites	57 (20.4)	14 (11.6)	10 (11.6)	33 (45.8)
**Medical characteristics, n (%)**					
	Duration of treatment<9 years	93 (33.3)	32 (26.4)	22 (25.6)	39 (54.2)
	Receiving treatments other than antiretroviral	123 (44.1)	65 (53.7)	40 (46.5)	18 (25.0)
	History of illegal drug use	55 (19.7)	18 (14.9)	9 (10.5)	28 (38.9)
**Internet and social media, n (%)**					
	Used the internet in the last 12 months to look for information or advice on health or wellness	154 (55.2)	64 (52.9)	32 (37.2)	58 (80.6)
	Changed the way they attend to their health/wellness after these searches	74 (26.5)	31 (25.6)	5 (5.8)	38 (52.8)
	Possess a social media account (eg, Facebook, Twitter)	174 (62.4)	55 (45.5)	47 (54.7)	72 (100.0)
	No longer as trusting after confidentiality problems	66 (23.7)	21 (17.4)	17 (19.8)	28 (38.9)
**App use, n (%)**					
	Currently use mobile apps for monitoring physical activity	45 (16.1)	8 (6.6)	8 (9.3)	29 (40.3)
	Would be willing to use an app if it was recommended by a physician	199 (71.3)	94 (77.7)	41 (47.7)	64 (88.9)
	Would be willing to use an app if it was recommended by an associate	51 (18.3)	13 (10.7)	6 (7.0)	32 (44.4)
	Would be willing to use an app if they could manage on their own	102 (36.6)	35 (28.9)	50 (58.1)	17 (23.6)
	Think an ideal app should help follow adverse effects of medical drugs	208 (74.6)	110 (90.9)	41 (47.7)	57 (79.2)
	Think an ideal app should help follow vaccinations	212 (76.0)	107 (88.4)	45 (52.3)	60 (83.3)
	Think an ideal app should help get in touch with other patients	88 (31.5)	46 (38.0)	16 (18.6)	26 (36.1)
	Trust an app more than a health care professional	26 (9.3)	19 (15.7)	2 (2.3)	5 (6.9)
**Connected objects, n (%)**					
	Possess a connected object	61 (21.9)	16 (13.2)	11 (12.8)	34 (47.2)
	Could be persuaded to have connected objects and use them if medical insurance schemes reduced their contributions as an incentive	88 (31.5)	40 (33.1)	10 (11.6)	38 (52.8)

^a^MSM: men who have sex with men.

**Table 5:** Comparison of the three groups of people living with HIV obtained by mixed unsupervised classification.

Characteristic		Total (N=279)	Group 1 (n=121)	Group 2 (n=86)	Group 3 (n=72)
**Telemedicine, n (%)**					
	In favor of consultations by video conference	166 (59.5)	84 (69.4)	16 (18.6)	66 (91.7)
	Would prefer to use distance consultation to get a new prescription for treatment	207 (74.2)	95 (78.5)	45 (52.3)	67 (93.1)
	Would prefer to use distance consultation to consult for health problems that seem minor (eg, sore throat, cold)	123 (44.1)	63 (52.1)	15 (17.4)	45 (62.5)
	Would prefer to use distance consultation to monitor evolution of their HIV infection	92 (33.0)	49 (40.4)	8 (9.3)	35 (48.6)
	Think having a free internet terminal in the medical unit where they can enter data directly into their medical files before consultation would be a good thing	124 (44.4)	73 (60.3)	11 (12.8)	40 (55.6)
**Collection of personal data, n (%)**					
	Think their personal data might be misused	167 (59.9)	65 (53.7)	63 (73.3)	39 (54.1)
	Think the law adequately oversees the collection and use of personal data	99 (35.5)	54 (44.6)	11 (12.8)	34 (47.2)
	Think artificial intelligence will speed progress towards more individualized diagnosis and treatment	159 (57.0)	88 (72.7)	19 (22.1)	52 (72.2)
	Would like to have health digital safe space on a dedicated site hosted by a health data organization	94 (33.7)	41 (33.9)	16 (18.6)	37 (51.4)
**eHealth, n (%)**					
	Think the development of eHealth is a good thing	197 (70.6)	109 (90.1)	21 (24.4)	67 (93.1)
	Think the development of eHealth would be efficient for improving coordination among different health care practitioners	226 (81.0)	104 (86.0)	52 (60.4)	70 (97.2)
	Think the development of eHealth would be efficient for reducing travel	135 (48.4)	69 (57.0)	14 (16.3)	52 (72.2)
	Think the development of eHealth would be efficient for servicing medically deprived areas	157 (56.3)	74 (61.2)	34 (39.5)	49 (68.1)
	Think the development of eHealth would be efficient for reducing the social Security burden	125 (44.8)	61 (50.4)	19 (22.1)	45 (62.5)

#### Group 1: eHealth Believers

The 121 PLHIV grouped as “eHealth believers” were most often aged over 60 years, and were more likely to be receiving treatments other than antiretroviral drugs. Compared to the patients in the other two groups, fewer of the patients in Group 1 had Facebook, Twitter, Instagram, LinkedIn, or other social media accounts, and fewer had downloaded mobile apps for wellness, health, or physical activity monitoring. More of these patients agreed that an ideal app should first show their vaccinations and adverse effects to their drugs, and then provide help with overall psychological wellness, monitor their physical state, and show their biological HIV status, history of HIV-related biology reports, and history of antiretroviral treatments. More of the patients in this group were also in contact with other individuals with the same interests, and trusted an app more than a health care professional. Many of them agreed that the collection of personal health data would increase in the coming years, that this trend would help to improve the quality of patient care and follow up, and that it was an acceptable price to be paid to gain benefits from the apps. Many feared that their personal health data might be misused, but more of them thought that artificial intelligence would progress toward more individualized diagnosis and treatments. In general, the great majority agreed that eHealth was a good thing, that it was efficient for coordination among health care professionals, and for monitoring the evolution of their HIV infection more regularly and more rapidly, allowing them greater autonomy, improved medical care and treatment, and reducing the societal burden.

#### Group 2: Technology Skeptics

The 86 PLHIV classified as “technology skeptics” were more often women, and more frequently had at least one child compared to patients in the other two groups. Fewer had smartphones, but more of these patients used mobile apps they had chosen themselves and not on the advice of a friend, physician, pharmacist, associate, or another patient. Fewer were comfortable with technology or trusted it. More had no time to use apps, were skeptical about their scientific value, and found them stressful. Fewer would be persuaded to use connected objects by their medical insurance scheme offering lower contributions. More of these patients were unwilling to share their data, including with their physician, and were less often in favor of distance consultations for serious health problems, to address an intimate subject, renew a prescription, ask for a medical certificate or for advice, monitor their HIV evolution, or get an emergency consultation. More of them would not want to use an internet terminal at their care facility to enter data directly into their hospital files. More of them were worried about the collection of personal health information and felt that the law did not oversee such collection to a sufficient degree. Concerning the digital safety of health information, more of these patients preferred hosting of apps by a health insurance organization, and considered the development of eHealth as a bad trend overall.

#### Group 3: Internet Adopters

The 72 PLHIV classified as “internet adopters” were most often aged under 49 years, male, and men who have sex with men, most of whom had higher education and stable jobs. Patients in this group were more likely to frequent bars and sex clubs and to use dating sites with geolocating apps, and to have consumed illegal drugs. They had been infected with HIV for less than 12 years, and had been receiving treatment for less than 9 years.

The main characteristic of this group was that they are regular internet users, including following an association connected with HIV via social media, and using mobile apps for wellness, health, or physical activity monitoring. More of them agreed that an ideal app should help them monitor their physical activity and keep track of their appointments. Fewer thought the apps intruded too much into their lives, and more used connected objects. Compared to the other two groups, more of these patients were willing to communicate their results to their general practitioner and their specialist physician, and more of them had already used email for such contact. Concerning the digital safety of health information, more of them would choose a health data host. In general, more of these patients considered that eHealth was useful overall, especially for monitoring their health indicators and reducing travel.

### Physician Profiles on eHealth Perceptions

#### Overall Response

**Figure 3:**
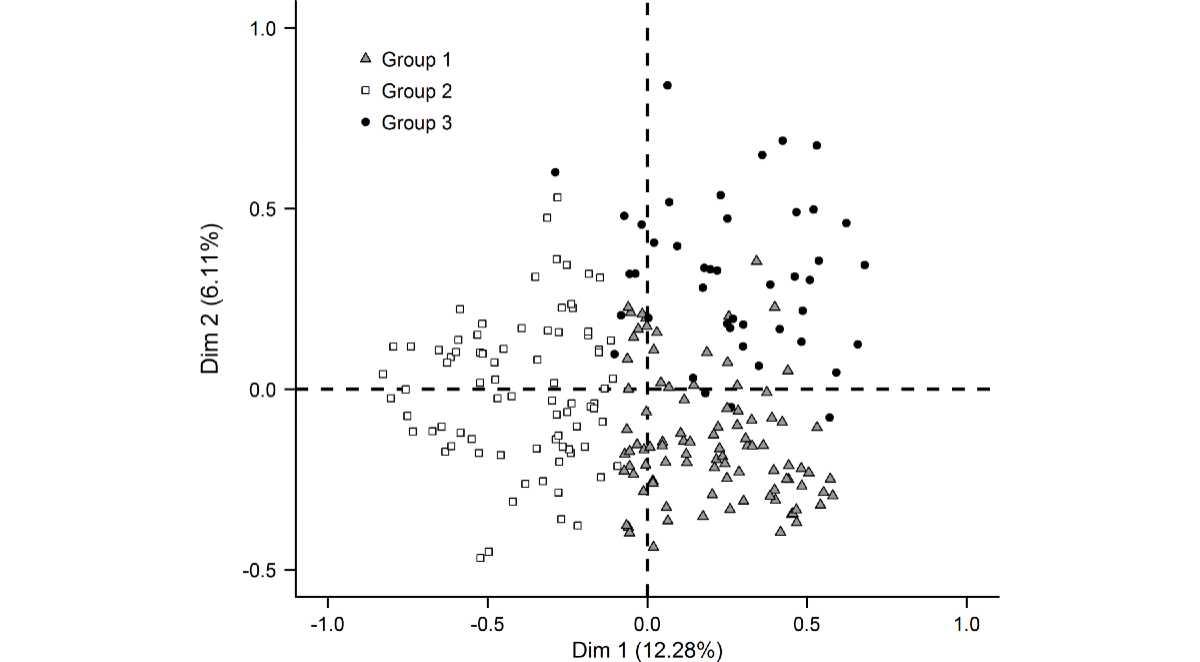
Factorial analysis of physicians by sociodemographic and medical characteristics, and their answers to 53 questions concerning eHealth: searching for information on the internet and social media, collection of digitized personal information and telemedecine, and mHealth apps and connected objects for health/wellness.

The analysis included 219 physicians for whom no data were missing among the variables selected for analysis. Multiple correspondence analysis identified three groups (G): G1 (95/219, 43.4%), termed “strongly confident in eHealth;” G2 (80/219, 36.5%), termed “strongly opposed to eHealth;”, and G3 (44/219, 20.1%), termed “open to eHealth” (Figure 3).

The eHealth perception characteristics of the physicians are summarized in Multimedia Appendix 2.

#### Group 1: Strongly Confident in eHealth

The 95 physicians “strongly confident in eHealth” were more often themselves users of wellness/health apps and more often reported having patients that actively use mobile apps. Compared to the other two groups, more of the physicians in Group 1 stated that they would like to see connected objects for health become generalized, and think that connected objects should be financed by health insurance schemes and that their use would reduce health costs. In addition, since the advent of new computerized medical information systems in their hospitals, these physicians largely agreed that they have better tools to work with, that diagnostic aid is a reality, and medical decision making is facilitated. More of them use computer-assisted prescribing, consider patient safety is improved, and that the eHealth provision favors a transfer of skills among professionals. More of them think that eHealth facilitates communication among physicians and are keen to conduct consultations by video conference.

#### Group 2: Strongly Opposed to eHealth

The 80 physicians “strongly opposed to eHealth” were more likely not to know their patients’ habits concerning connected objects. More of them agreed that eHealth challenges the confidentiality of medical information, consider a risk for data security and quality, and that eHealth destroys the care system. More of them disagreed that eHealth will reduce the number of consultations, will improve the quality and efficacy of patient care, or embody technical progress. More of the physicians in this group were against the collection of personal health information, and disagreed that it will improve patient care and follow up. More of these physicians were also mistrustful and uneasy about the potential uses of personal information, and did not consider that the law appropriately oversees the collection of health data.

#### Group 3: Open to eHealth

The 44 physicians “open to eHealth” were more often infectious disease specialists compared to the specialties of physicians in the other two groups. More of these physicians considered that mobile medical apps are useful for informing and educating their patients, assisting clinical decision making, facilitating online entering of data in medical files, and improving patient monitoring. More of them considered that connected objects enable better quality of data for care, and that eHealth facilitates communication with paramedics. More of them asserted that the development of eHealth is a good trend overall, because it will be efficient in improving coordination among health care professionals, following patients’ health indicators more closely, improving the quality of care and treatment, servicing medically deprived areas, and making more data available for public health.

### Association Between Physician and Patient Profiles

There was no link between the three physician and three patient groups (*P*=.37).

## Discussion

### Principal Findings

This is the first study to examine the patterns of use, benefits, as well as perceived obstacles and challenges for eHealth in PLHIV and their physicians using a detailed questionnaire on different aspects of eHealth, including retrieval of information on the internet and social media, mobile apps, and connected objects, along with the use of telemedicine and the collection of digitized personal health information in France and elsewhere.

Our findings revealed three distinct clusters of patients: (i) those for whom eHealth is part of a connected lifestyle; (ii) those who mistrust technology, although they are more averse to technology in general than to eHealth specifically; and (iii) those keen to adopt eHealth because they see it as a benefit for their health, and for whom eHealth does not represent any risk. Three clusters were also found for the physicians: (i) those strongly opposed to eHealth (resisters), (ii) those who believe in eHealth (enthusiasts), and (iii) those who are open to eHealth, and who rise to the challenge. This third group overlaps the second to some extent.

### Strengths and Limitations

It should be emphasized that some PLHIV are logically exempt from hospital surveys, including those diagnosed but without follow up and those followed outside a hospital setting, whose number is increasing. In addition, recruitment was conducted on a voluntary basis and those unable to speak/read French or unable to complete a questionnaire were excluded. Among those eligible, PLHIV who did not have access to the internet or who were not comfortable with computers, and were thus likely technology skeptics, did not agree to participate in the study. Thus, if 838/1233 (67.96%) PLHIV agreed to participate, only 325/838 (38.8%) of them answered all of the questions, which could impact the representativeness of the sample. However, the design of this survey, conducted over a single week in 51 care units in France and its overseas departments, and the fact that the characteristics of those who answered all of the questions were not widely different from those reported for the general PLHIV population in France [18] entails the soundness of results, besides the rate of 86% of physicians included. In addition, these PLHIV were only 2 years older on average than the whole French PLHIV population, and this difference is not generational. Moreover, 30% is the usual rate of responses to online surveys [19].

The lifelong drug therapy requiring regular follow-up visits with laboratory tests is well documented from this survey. The high prevalence of multiple clinical comorbidities with concomitant polypharmacy necessitates effective interdisciplinary care, but also access to web-based information or connection of apps enabling PLHIV to monitor their health [20].

### Implications

Overall, our study found that 55% of the PLHIV had already searched for information on the internet. This finding adds to a study published in 2013 that found 49% of people affected by a chronic illness or serious condition consulted the internet for health-related questions [21]. The fact that online searches could enable self-diagnosis or self-medication has already been discussed elsewhere [22]. In our survey, 74 of 154 (48.1%) PLHIV respondents changed the way they cared for their health after relevant internet searches. Participating in an association connected with HIV was less frequent (9%), probably because of the discrimination that PLHIV might be subject to, which is in contrast to patients with other chronic health disorders, 30% of whom discuss or exchange information about their illnesses [23].

The data presented herein suggest that an ideal app for PLHIV in France would be one that enables follow up of vaccinations and monitoring of physical health conditions linked to HIV. The purpose of such an app would be to “communicate better” with the patient’s physician, extending the first findings of the Emerge project [24]. The questions then arise as to how this communication will be managed for physicians, how it will be integrated into the patient’s care path, and who will pay for its implementation. The responses of physicians to these issues did not differ across groups: 42% of the physicians saw no use for mobile apps, whereas 41% thought they might be useful for their patients to keep track of their appointments. An Odoxa survey conducted in 2015 found that 29% of patients regularly used common connected objects, and only 5% had been advised by their physician to use connected technology [25].

Interestingly, 22% of PLHIV and 11% of physicians who stated that some of their patients reported using connected objects emphasized that digital technologies in medical practice were still in their infancy. One reason for this lack of eager promotion may be related to controversial reports [26] on the positive consequences of such technologies for morbidity and mortality.

In January 2019, 52% of French respondents were in favor of distance consultations [27]. The percentage was higher among PLHIV (60%) in the present study. However, serious misgivings about data collection remain: more than one third of respondents, both PLHIV and physicians, considered that the law did not oversee such data collection to a sufficient degree. The security and privacy concerns about both the devices that collect data and the systems in which these data are stored are prominent, and thus require more specific regulations [28].

In our study, the PLHIV and physicians most often using eHealth were those who also generally used the internet frequently. Those who used eHealth least were those who were averse to technology in general. Some researchers in this field [29] have indicated a continuum from technophiles to technophobes representing a continuum of idealization to skepticism toward eHealth. Rio del Carral and colleagues [30] recently published the results of a survey conducted among a population attending a public fair on health in Lausanne. In 2018, most of the individuals surveyed (55%) expressed a reluctance to possess connected objects and physical health apps because they feared misuse of shared data. The study further showed that these tools reinforced the existing habits and practices of some people (eg, physical activity and diet), rather than being “useful” for those who did not yet have a “healthy” lifestyle, indicating a health technology divide among the population studied. It is well-established that health-related and literacy-related disparities should be minimized for eHealth to be widely accepted and adopted [20]. Certainly, we observed a divide of this sort in our study, but it is noteworthy that a third profile appeared within both PLHIV and physicians, including PLHIV attentive to eHealth for improving their condition and physicians who perceive a benefit for their patients or their practice. Only 31% of patients and 36% of physicians remained respectively mistrustful of or hesitant about using eHealth. With a fine-scale analysis of the sociodemographic and medical characteristics of respondents, we can better describe the characteristics and representations of people that fall somewhere between the extremes of “in favor” and “against,” between enhancement by technology and enfeeblement and enslavement by technology [31]. This intermediate group comprised PLHIV “eHealth believers” who were older, with more comorbidities, and who viewed eHealth as a response to their multiple health disorders and the more complex care path they were offered. Their responses emphasize that they were not especially technophiles or attentive to their health, and while perceiving the benefits of eHealth, they did not easily fit along a technophile-technophobe continuum. Compared to the “internet adopters,” fewer of the “eHealth believers” wanted to have consultations by video conference, especially for an intimate matter. It is noteworthy that such a group of older “eHealth believers” has not yet been identified in similar studies focused on other health conditions, in particular cardiac illnesses [32].

Méadel and Akrich [33] reported that studies documenting how physicians interact with “informed patients” show polarized reactions. Some studies found that patients who consulted the internet were poorly informed (overanxious and overdemanding), which prolonged consultations because the information brought by the patient had to be discussed and explained. Others, often more familiar with this tool, considered such consultation to be positive and likely to help patients become more active in their own health care. Here, we describe an additional group of physicians, “open to eHealth,” who were most often infectious disease specialists, and were neither idealists nor skeptics, but saw the possibilities of eHealth for developing greater autonomy among their patients even though not all information can be entrusted to machines, similar to the argument put forth by Besnier [34]. In other words, some patients and physicians seem to pay greater attention to eHealth projects than other online initiatives; they consider eHealth as a contemporary “civilizational” tool designed to help users and improve their health condition or mode of practice [35]. In that respect, eHealth could play the part of a “mediator” [36].

It is interesting that the debate on eHealth extends beyond the scope of individual medical practice. Although PLHIV who were “adopters” and “believers” with regard to eHealth both recognized the benefit of eHealth in terms of coordination among different health care professionals, only the physicians “open to eHealth” favored overcoming the opposition between medicine and public health. The absence of any statistical link between the patient and physician groups suggests that little cogent discussion on the subject was taking place during consultations.

The coordinated adoption of eHealth by PLHIV and physicians may be the next step following the dissemination of our results within the French society in the fight against HIV/AIDS. Once physicians and patients share the same perceptions of eHealth, it is possible that the common use of tools accepted by both parties will lead to improved care.

### Conclusion

Given the successful scale up of antiretroviral therapy globally, eHealth apps have the potential to transform HIV care beyond viral suppression in terms of comorbidity management and patient-reported outcome [37]. Our study shows that 26% of PLHIV and 43% of physicians are eHealth enthusiasts, while 31% and 37% are skeptics. For the latter group, there is a need to scale down literacy-related disparities; enforce regulations that will reduce concerns about security, privacy, and sharing personal data; as well as to ensure that physicians assisted by better hospital management are involved in the promotion of apps and connected objects in telemedicine. However, a third profile overcoming these challenges appeared in both PLHIV (43%), who were often older and attentive to eHealth for improving their health condition, and physicians (20%), who find benefits of eHealth for patients or their own practice, although without a direct link between the two groups. It seems necessary for both PLHIV and physicians to address eHealth apps during consultations, which could lead to improvement in care and to a reduction of the cost of care pathways.

## Appendix

Multimedia Appendix 1 Comparison of the three groups of people living with HIV obtained by mixed unsupervised classification.

Multimedia Appendix 2 Comparison of the three physician groups obtained by mixed unsupervised classification.

## Abbreviations

CHU: Centre Hospitalier Universitaire
eHealth: electronic health
EPICES: Evaluation of Precarity and Inequalities in Health Examination Centers
GDP: gross domestic product
MSM: men who have sex with men
PLHIV: people living with HIV
REDCap: Research Electronic Data Capture
VAS: visual analog scale
