# Supplementary material for: Adoption and Attitudes of eHealth Among People Living With HIV and Their Physicians: Online Multicenter Questionnaire Study
Source: JMIR Mhealth Uhealth. 2020 Apr 15;8(4):e16140. doi: 10.2196/16140 (PMC7191352; doi:10.2196/16140)
Supplement: Multimedia Appendix 1 [file mhealth_v8i4e16140_app1.docx]

**Appendix 1 Table S3. Comparison of the three PLHIV groups obtained by mixed unsupervised classification.**

|  | **Total**  ***N* = 279**  ***n* (%)** | **Group 1 *n* = 121**  ***n* (%)** | | **Group 2 *n* = 86**  ***n* (%)** | | **Group 3 *n* = 72**  ***n* (%)** |
| --- | --- | --- | --- | --- | --- | --- |
| **Sociodemographic characteristics** |  |  | |  | |  |
| Age |  |  | |  | |  |
| less than 49 years | 106 (38) | 33 (27) | | 26 (30) | | 47 (65) |
| between 50 and 59 years | 94 (34) | 40 (33) | | 35 (41) | | 19 (26) |
| more than 60 years | 79 (28) | 48 (40) | | 25 (29) | | 6 (8) |
| Male | 199 (71) | 90 (74) | | 51 (59) | | 58 (81) |
| MSM | 120 (43) | 53 (44) | | 21 (24) | | 46 (64) |
| At least one child | 119 (43) | 50 (41) | | 48 (56) | | 21 (29) |
| Higher education | 184 (66) | 73 (60) | | 51 (59) | | 60 (83) |
| Stable job | 130 (47) | 45 (37) | | 40 (47) | | 45 (63) |
| Frequent geolocating dating sites | 57 (20) | 14 (12) | | 10 (12) | | 33 (46) |
| **Medical characteristics** |  |  | |  | |  |
| Duration of HIV infection less than 12 years | 98 (35) | 30 (25) | | 26 (30) | | 42 (58) |
| Duration of treatment less than 9 years | 93 (33) | 32 (26) | | 22 (26) | | 39 (54) |
| Receiving treatments other than antiretroviral | 123 (44) | 65 (54) | | 40 (47) | | 18 (25) |
| Have already consumed illegal drugs | 55 (20) | 18 (15) | | 9 (10) | | 28 (39) |
| **Internet and social media** |  |  | |  | |  |
| Have used the internet in the last 12 months to look for information or advice on health or wellness | 154 (55) | 64 (53) | | 32 (37) | | 58 (81) |
| Remember the subject of their last internet search | 111 (40) | 49 (41) | | 22 (26) | | 40 (56) |
| Have changed the way they attend to their health/wellness after these searches. | 74 (27) | 31 (26) | | 5 (6) | | 38 (53) |
| Possess a social media account (Facebook, Twitter, etc.) | 174 (62) | 55 (45) | | 47 (55) | | 72 (100) |
| Follow an association connected with HIV via social media | 24 (9) | 3 (2) | | 3 (3) | | 18 (25) |
| No longer as trusting after confidentiality problems | 66 (24) | 21 (17) | | 17 (20) | | 28 (39) |
| Possess a smartphone | 247 (89) | 105 (87) | | 71 (83) | | 71 (99) |
| Possess a tablet computer | 112 (40) | 42 (35) | | 29 (34) | | 41 (57) |
| **Applications** |  |  | |  | |  |
| Have already downloaded and used mobile applications for health/wellness/monitoring of physical activity | 74 (27) | 12 (10) | | 16 (19) | | 46 (64) |
| Currently use mobile applications for wellness | 26 (9) | 0 (0) | | 2 (2) | | 24 (33) |
| Currently use mobile applications for monitoring physical activity | 45 (16) | 8 (7) | | 8 (9) | | 29 (40) |
| Would be ready to use an application if it was recommended by: |  |  | |  | |  |
| 1. A friend | 43 (15) | 14 (12) | | 4 (5) | | 25 (35) |
| 1. A physician | 199 (71) | 94 (78) | | 41 (48) | | 64 (89) |
| 1. A pharmacist | 68 (24) | 23 (19) | | 9 (10) | | 36 (50) |
| 1. An association | 51 (18) | 13 (11) | | 6 (7) | | 32 (44) |
| 1. Another patient | 36 (13) | 8 (7) | | 5 (6) | | 23 (32) |
| 1. Could manage on their own | 102 (37) | 35 (29) | | 50 (58) | | 17 (24) |
| Think an ideal application should help them: |  |  |  | |  | |
| 1. Follow psychological wellness | 203 (73) | 109 (90) | 39 (45) | | 55 (76) | |
| 1. Follow physical activity | 204 (73) | 104 (86) | 37 (43) | | 63 (88) | |
| 1. Follow physical health condition | 211 (76) | 110 (91) | 39 (45) | | 62 (86) | |
| 1. Follow biological test results for HIV | 190 (68) | 102 (84) | 35 (41) | | 53 (74) | |
| 1. Follow history of antiretroviral treatments | 189 (68) | 104 (86) | 31 (36) | | 54 (75) | |
| 1. Follow adverse effects of medical drugs | 208 (75) | 110 (91) | 41 (48) | | 57 (79) | |
| 1. Follow vaccinations | 212 (76) | 107 (88) | 45 (52) | | 60 (83) | |
| 1. Keep track of their medical appointments | 199 (71) | 100 (83) | 36 (42) | | 63 (88) | |
| 1. Get in touch with other persons | 88 (32) | 46 (38) | 16 (19) | | 26 (36) | |
| Are comfortable with technology | 210 (75) | 97 (80) | 46 (53) | | 67 (93) | |
| Trust technology | 169 (61) | 86 (71) | 27 (31) | | 56 (78) | |
| Have the time to use applications | 187 (67) | 81 (67) | 41 (48) | | 65 (90) | |
| Have enough memory in their telephone | 151 (54) | 61 (50) | 30 (35) | | 60 (83) | |
| Believe in the scientific value of the applications | 139 (50) | 73 (60) | 12 (14) | | 54 (75) | |
| Trust an application more than a healthcare professional | 26 (9) | 19 (16) | 2 (2) | | 5 (7) | |
| Think applications are stressful and cause anxiety | 107 (38) | 50 (41) | 41 (48) | | 16 (22) | |
| Think applications intrude too much into their life | 158 (57) | 75 (62) | 54 (63) | | 29 (40) | |
| Think there are hidden costs | 107 (38) | 55 (45) | 35 (41) | | 17 (24) | |
| **Connected objects** |  |  |  | |  | |
| Possess a connected object | 61 (22) | 16 (13) | 11 (13) | | 34 (47) | |
| Could be persuaded to have connected objects and use them if medical insurance schemes reduced their contributions as an incentive. | 88 (32) | 40 (33) | 10 (12) | | 38 (53) | |
| Would communicate results obtained from connected objects to: |  |  |  | |  | |
| 1. Their general practitioner | 195 (70) | 89 (74) | 48 (56) | | 58 (31) | |
| 1. Their specialist physician | 214 (77) | 97 (80) | 55 (64) | | 62 (86) | |
| 1. Their pharmacist | 33 (12) | 18 (15) | 4 (5) | | 11 (15) | |
| 1. Medical insurance schemes | 42 (15) | 19 (16) | 7 (8) | | 16 (22) | |
| 1. No-one | 46 (16) | 12 (10) | 27 (31) | | 7 (10) | |
| **Telemedicine** |  |  |  | |  | |
| Are in favour of consultations by videoconference | 166 (60) | 84 (69) | 16 (19) | | 66 (92) | |
| Would prefer to use distance consultation to: |  |  |  | |  | |
| 1. Get a new prescription for treatment | 207 (74) | 95 (79) | 45 (52) | | 67 (93) | |
| 1. Ask for a medical certificate | 182 (65) | 74 (61) | 48 (56) | | 60 (83) | |
| 1. Ask for medical advice | 168 (60) | 79 (65) | 32 (37) | | 57 (79) | |
| 1. Consult for health problems that seem minor (sore throat, cold, etc.) | 123 (44) | 63 (52) | 15 (17) | | 45 (63) | |
| 1. Monitor the evolution of their HIV infection | 92 (33) | 49 (41) | 8 (9) | | 35 (49) | |
| 1. Talk about an intimate or sensitive health problem | 53 (19) | 23 (19) | 4 (5) | | 26 (36) | |
| 1. Get an emergency consultation | 114 (41) | 60 (50) | 18 (21) | | 36 (50) | |
| 1. Consult for health problems that seem serious | 51 (18) | 28 (23) | 5 (6) | | 18 (25) | |
| Think distance consultation is a step forward | 217 (78) | 112 (93) | 35 (41) | | 70 (97) | |
| Have already contacted their general practitioner by email | 36 (13) | 17 (14) | 4 (5) | | 15 (21) | |
| Have already contacted their HIV specialist physician by email | 77 (28) | 30 (25) | 18 (21) | | 29 (40) | |
| Have never contacted any healthcare professional by email | 174 (62) | 77 (64) | 65 (76) | | 32 (44) | |
| Think having a free internet terminal in the medical unit where they can enter data directly into their medical files before consultation would be a good thing | 124 (44) | 73 (60) | 11 (13) | | 40 (56) | |
| **Collection of personal data** |  |  |  | |  | |
| Think the collection of personal health information will increase in the years to come | 229 (82) | 112 (93) | 48 (56) | | 69 (96) | |
| Think this trend is improving the quality of care and patient follow-up | 187 (67) | 102 (84) | 20 (23) | | 65 (90) | |
| Are worried about this trend | 128 (46) | 47 (39) | 58 (67) | | 23 (32) | |
| Think use of personal data is the price they have to pay to gain benefit from health applications | 147 (53) | 77 (64) | 18 (21) | | 52 (72) | |
| Would be ready to pay a subscription for their data to be better protected | 53 (19) | 18 (15) | 8 (9) | | 27 (38) | |
| Think their personal data might be misused | 167 (60) | 65 (54) | 63 (73) | | 39 (54) | |
| Think the law adequately oversees the collection and use of personal data | 99 (35) | 54 (45) | 11 (13) | | 34 (47) | |
| Think artificial intelligence will speed progress towards more individualised diagnosis and treatment | 159 (57) | 88 (73) | 19 (22) | | 52 (72) | |
| Would like to have a health ‘digital safe’ on a dedicated site hosted by a health data organisation | 94 (34) | 41 (34) | 16 (19) | | 37 (51) | |
| **e-health** |  |  |  | |  | |
| Think the development of e-health is a good thing | 197 (71) | 109 (90) | 21 (24) | | 67 (93) | |
| Think the development of e-health would be efficient for: |  |  |  | |  | |
| 1. Improving coordination among different healthcare practitioners | 226 (81) | 104 (86) | 52 (60) | | 70 (97) | |
| 1. More regular and faster monitoring of their HIV infection | 124 (44) | 72 (60) | 7 (8) | | 45 (63) | |
| 1. Better monitoring of health indicators | 109 (39) | 61 (50) | 7 (8) | | 41 (57) | |
| 1. Reducing their travel | 135 (48) | 69 (57) | 14 (16) | | 52 (72) | |
| 1. Giving them greater autonomy | 87 (31) | 44 (36) | 7 (8) | | 36 (50) | |
| 1. Improving the quality of medical care and treatment | 115 (41) | 62 (51) | 12 (14) | | 41 (57) | |
| 1. Servicing medically deprived areas | 157 (56) | 74 (61) | 34 (40) | | 49 (68) | |
| 1. Reducing the Social Security burden | 125 (45) | 61 (50) | 19 (22) | | 45 (63) | |
