# Supplementary material for: Adoption and Attitudes of eHealth Among People Living With HIV and Their Physicians: Online Multicenter Questionnaire Study
Source: JMIR Mhealth Uhealth. 2020 Apr 15;8(4):e16140. doi: 10.2196/16140 (PMC7191352; doi:10.2196/16140)
Supplement: Multimedia Appendix 2 [file mhealth_v8i4e16140_app2.docx]

**Appendix 2 Table S4. Comparison of the three physician groups obtained by mixed unsupervised classification.**

| **Variable** | **Total *N* = 219**  ***n* (%)** | **Group 1 *n* = 95**  ***n* (%)** | **Group 2 *n* = 80**  ***n* (%)** | **Group 3 *n* = 44**  ***n* (%)** |
| --- | --- | --- | --- | --- |
| **Individual characteristics** |  |  |  |  |
| Speciality infectiology | 157 (72) | 69 (73) | 50 (63) | 38 (86) |
| Since new computerised medical information systems have been set up in their hospital: |  |  |  |  |
| 1. They have better tools to work with | 141 (64) | 76 (80) | 37 (46) | 28 (64) |
| 1. Assisted diagnosis is a reality | 82 (37) | 52 (55) | 13 (16) | 17 (39) |
| 1. Medical decision-making has been made easier | 93 (42) | 59 (62) | 18 (23) | 16 (36) |
| 1. They use the assisted prescription software | 135 (62) | 68 (72) | 40 (50) | 27 (61) |
| 1. Patient safety is improved | 131 (60) | 73 (77) | 31 (39) | 27 (61) |
| 1. There is a transfer of skills among healthcare professionals | 90 (41) | 57 (60) | 13 (16) | 20 (45) |
| **Applications** |  |  |  |  |
| They use applications for wellness | 76 (35) | 41 (43) | 18 (23) | 17 (39) |
| Have patients who use applications to monitor their health | 91 (42) | 47 (49) | 26 (33) | 18 (41) |
| Think mobile medical applications enable: |  |  |  |  |
| 1. Better patient information and education | 169 (77) | 83 (87) | 46 (58) | 40 (91) |
| 1. Help with clinical decision making | 88 (40) | 45 (47) | 21 (26) | 22 (50) |
| 1. Online entry of data in medical files | 46 (21) | 27 (28) | 5 (6) | 14 (32) |
| 1. Better patient monitoring | 85 (39) | 47 (49) | 13 (16) | 25 (57) |
| **Connected objects** |  |  |  |  |
| Have patients who use connected objects | 24 (11) | 17 (18) | 3 (4) | 4 (9) |
| Are in favour of generalised use of connected objects for health | 133 (61) | 79 (83) | 19 (24) | 35 (80) |
| Think these objects should be paid for by medical insurance schemes | 72 (33) | 44 (46) | 13 (16) | 15 (34) |
| Think these objects can reduce healthcare costs | 94 (43) | 52 (55) | 22 (28) | 20 (45) |
| Think these objects can improve the quality of data for medical care | 142 (65) | 64 (67) | 38 (48) | 40 (91) |
| **Telemedicine** |  |  |  |  |
| Would like videoconference consulting | 76 (35) | 48 (51) | 10 (13) | 18 (41) |
| **Collection of personal information** |  |  |  |  |
| Think the collection of personal health information will help to improve the quality of patient care and follow-up | 135 (62) | 81 (85) | 18 (23) | 36 (82) |
| Are concerned about the use of personal health data | 155 (71) | 61 (64) | 67 (84) | 27 (61) |
| Think use of personal data is the price they have to pay to gain benefit from health applications | 75 (34) | 35 (37) | 18 (23) | 22 (50) |
| Think personal data might be misused | 119 (54) | 35 (37) | 65 (81) | 19 (43) |
| Are personally against collecting this type of data | 54 (25) | 12 (13) | 37 (46) | 5 (11) |
| Think the law adequately oversees the collection and use of health data | 75 (34) | 47 (49) | 12 (15) | 16 (36) |
| **e-health** |  |  |  |  |
| Think the development of e-health is a good thing | 176 (80) | 88 (93) | 44 (55) | 44 (100) |
| Think the development of e-health would be efficient for: |  |  |  |  |
| 1. Improving coordination among different healthcare professionals | 49 (22) | 2 (2) | 7 (9) | 40 (91) |
| 1. Better follow-up of patient health indicators | 34 (16) | 1 (1) | 6 (8) | 27 (61) |
| 1. Improving the quality of medical care and treatment | 35 (16) | 2 (2) | 2 (3) | 31 (70) |
| 1. Servicing medically deprived areas | 18 (8) | 0 (0) | 2 (3) | 16 (36) |
| 1. Increasing data for public health | 32 (15) | 0 (0) | 4 (5) | 28 (64) |
| Think e-health facilitates the consultation of databases | 151 (69) | 72 (76) | 48 (60) | 31 (70) |
| Think e-health facilitates communication with physicians | 115 (53) | 61 (64) | 28 (35) | 26 (59) |
| Think e-health facilitates communication with paramedics | 79 (36) | 42 (44) | 14 (18) | 23 (52) |
| Think e-health acts: |  |  |  |  |
| 1. Will reduce the number of consultations | 76 (35) | 43 (45) | 11 (14) | 22 (50) |
| 1. Will bring physicians economic benefits | 23 (11) | 14 (15) | 3 (4) | 6 (14) |
| 1. Challenge medical confidentiality | 94 (43) | 26 (27) | 51 (64) | 17 (39) |
| 1. Enable improved quality of patient care | 115 (53) | 71 (75) | 12 (15) | 32 (73) |
| 1. Enable greater efficacy of patient care | 131 (60) | 79 (83) | 20 (25) | 32 (73) |
| 1. Raise data protection issues | 151 (69) | 56 (59) | 65 (81) | 30 (68) |
| 1. Raise data quality issues | 117 (53) | 37 (39) | 58 (73) | 22 (50) |
| 1. Debase the medical care system | 57 (26) | 15 (16) | 38 (48) | 4 (9) |
| 1. Embody technological progress | 159 (73) | 87 (92) | 35 (44) | 37 (84) |
